# Comprehensive Analysis of Gene Signatures of m6ARNA Methylation Regulators in Lung Adenocarcinoma and Development of a Risk Scoring System

**DOI:** 10.1155/2022/7519838

**Published:** 2022-08-23

**Authors:** Chundi Gao, Huayao Li, Wenzhe Ma, Qiming Zhang, Cun Liu, Lijuan Liu, Jing Zhuang, Changgang Sun

**Affiliations:** ^1^College of First Clinical Medicine, Shandong University of Traditional Chinese Medicine, Jinan 250014, Shandong, China; ^2^College of Basic Medical, Shandong University of Traditional Chinese Medicine, Jinan 250014, Shandong, China; ^3^College of Chinese Medicine, Weifang Medical University, Weifang, China; ^4^State Key Laboratory of Quality Research in Chinese Medicine, Macau University of Science and Technology, Macau, China; ^5^Department of Experimental Research Center, China Academy of Chinese Medical Sciences, Beijing, China; ^6^Department of Oncology, Weifang Traditional Chinese Hospital, Weifang 261041, Shandong, China; ^7^Academy of Chinese Medical Sciences, Shandong University of Traditional Chinese Medicine, Qingdao, Shandong Province, China

## Abstract

The recent application of targeted immunotherapy has greatly improved the clinical outcomes of patients with lung adenocarcinoma (LUAD), but drug resistance continues to emerge, and to evaluate and to improve patient prognosis are arduous. The diagnostic and prognostic value of N6-methyladenosine (M6A) in LUAD has attracted increasing attention. We systematically studied correlations among important M6A methylation regulators, tumor mutational burden (TMB), and immune infiltration in clinical and sequencing data from the LUAD cohort of the cancer genome map (TCGA). The molecular subtype clusters 1 and 2 were identified by the consensus clustering of 16 M6A regulatory factors. Clinical prognosis, M6A regulatory factor expression, TMB, pathway enrichment, and immune cell infiltration significantly differed between clusters 1 and 2. Compared with other clinical traits, a prognostic risk score system constructed using the M6A regulatory factors HNRNPA2B1 and HNRNPC can serve as an independent prognostic method for LUAD, with higher predictive sensitivity and specificity. Risk scores were significantly higher for cluster 2 than 1, which was consistent with the trend towards a better prognosis in cluster 1. Overall, our findings revealed an important role of M6A methylation regulators in LUAD, and our risk scoring system involving these regulators might help to screen groups at high risk for LUAD and provide important theoretical bioinformatic support for evaluating the prognosis of such patients.

## 1. Introduction

Adenocarcinoma is the most prevalent histological subtype of primary lung cancer, and it is usually diagnosed at an advanced stage involving metastatic tumors [1]. Lung adenocarcinoma (LUAD) is one of the most aggressive and rapidly fatal tumor types, with an overall survival rate < 5 years. Understanding LUAD pathogenesis and treatment has recently advanced, and this is particularly important for the development of new cancer treatment strategies [2–4].

N6-methyladenosine is the most common chemical modification of messenger RNA (mRNA), and it is heritable and reversible. The biological functions of M6A are mediated through methylation by M6A methyltransferase (writer), demethylation by M6A demethylase (eraser), and recognition by M6A binding protein (reader), which affect mRNA splicing, conversion, and stability. Reversible M6A methylation alters the expression of genes without changing their nucleotide sequences and participates in a series of biological processes [5, 6]. Abnormal M6A methylation is closely associated with stem cell differentiation, immune responses, embryonic development, and microRNA editing [7]. Although RNA modification is not generally considered as a driving factor for cancer, accumulating evidence shows that M6A regulatory factors are closely associated with carcinogenic or malignant tumor suppressor functions, including proliferation, tumorigenesis, invasion, metastasis, and immune system escape [8, 9]. The m6A modification involves many aspects of LUAD; thus, it can serve as a biomarker for the prognosis of LUAD and participate in formation of the tumor microenvironment [10]. For example, methyltransferase like 3 (METTL3) promotes the growth, survival, and invasion of human lung cancer cells [11]. A decrease in YT521-B homology (YTH) domain-containing protein 2 (YTHDC2) is associated with poor clinical outcomes of LUAD [12]. The m6A modification is associated with clinical results and clinicopathological characteristics, and it can serve as an independent prognostic factor of LUAD, which has helped the development of more effective personalized treatment strategies [13].

The TMB is the latest independent predictor of the outcomes of treatment with immune checkpoint inhibitors across multiple tumor types and it has potential as a prognostic indicator for multiple tumor types [14, 15]. For example, hypermutant breast cancer is more likely to benefit from programmed cell death protein-1 (PD-1) inhibitors [16]. Tumor infiltrating cells are part of a complex microenvironment that promotes and/or regulates tumor development and growth. Depending on the cell type and its functional interaction, immune cells might play key roles in inhibiting tumors or supporting tumor growth [17]. The TMB is a negative predictive biomarker of overall survival (OS) in patients with advanced LUAD with EGFR mutations [18], B cell immune infiltration impacts the survival of patients with LUAD, and immune cell infiltration scores have significance for predicting the OS of patients with LUAD [19, 20]. Therefore, the TMB and immune cell infiltration are potential options for the treatment and prognosis of LUAD.

Here, we explored the role of N6-methylanisole (m6A) methylation-related mechanisms in the prevention and progression of LUAD and investigated the effects of m6A methylation on the biological function of LUAD. We characterized relationships between m6A and the TMB as well as tumor immune cell infiltration. We explored new methods of diagnosis, prognosis, and treatment of LUAD from the perspective of epigenetic modification to promote tumor progression.

## 2. Materials and Methods

### 2.1. Data Acquisition

The transcriptome, mutation, and clinical data of LUAD analyzed herein were obtained from The Cancer Genome Atlas (TCGA) database (https://cancergenome.nih.gov/) [21]. A transcriptional group contained 594 related samples comprising 59 normal and 535 LUAD samples. Combined with the clinical data, 490 LUAD samples were used for the grouping of training and verification sets and to construct a risk scoring system. We also selected 560 LUAD samples from TCGA mutation data to analyze target gene mutations and the TMB. Ethical approval was not required because the TCGA database is in the public domain.

### 2.2. Extraction and Analysis of M6A RNA Methylation Regulator

We extracted transcriptome expression data of 16 M6A RNA methylation regulatory factors from 594 LUAD samples and analyzed differences between normal and LUAD using R-packet EDGER. We categorized data from patients with LUAD into two subtypes using ConsensusClusterPlus software and then combined them with clinical information to evaluate M6A gene expression among LUAD subtypes and further clarify the biological characteristics of M6A regulatory factors in LUAD. Differences in pathway enrichment between the two clusters were further assessed using Gene Set Enrichment Analysis (GSEA) [22].

### 2.3. Characteristics of TMB and Immune Cell Infiltration among LUAD Subtypes

We counted the numbers of mutations including those that cause amino acid changes in related genes and then calculated the number of tumor mutations per Mb in each sample to generate corresponding TMB values. The LUAD samples were grouped according to 16 factors that regulate M6A RNA methylation to further compare differences in TMB levels between mutant and wild groups.

Based on the characterization of 22 tumor-infiltrating lymphocyte subsets, we evaluated the relative abundance of immune cells in the LUAD subtypes using the deconvolution algorithm CIBERSORT. Thereafter, relative contents of immune cells in the subtypes were visualized using the corrplot package, and correlations between immune cells were analyzed.

### 2.4. Construction of Risk Scoring System

We randomly assigned 490 samples from patients with LUAD to training (*n* = 245) and validation (*n* = 245) cohorts (proportion: 1 : 1) (Table 1). The M6A RNA methylation regulators related to the prognosis of LUAD were screened using the univariate Cox regression analyses, and then, an M6A-related LUAD risk score system was constructed using lasso and multivariate Cox regression.

Risk score equations were generated using the coefficients obtained using the multivariate Cox regression as

(1) $$\begin{array}{c} \text{Risk score}=\overset{N}{\underset{i=1}{\sum }}{\text{Exp}}_{i}\times \beta_{i}, \end{array}$$

where *β* represents the coefficient of M6A-related genes in the system and Exp represents the value of gene expression. Risk scores for each patient in the training and verification cohorts were calculated, and then, the patients were assigned to high- and low-risk groups using the median risk score as the cutoff. Survival rates were compared between the groups using the Kaplan-Meier curves.

### 2.5. Independent Predictive Analysis of Risk Scoring System

We further assessed whether the prognostic performance of the risk scoring system is independent of other clinical parameters. We obtained clinical data (including age, sex, stage, TNM stage, survival duration, and survival status) about the patients with LUAD and explored related independent prognoses using the univariate and multivariate Cox regression analyses. Statistical significance was set at *P* < 0.05.

## 3. Results

### 3.1. Extraction and Difference Analysis of M6A RNA Methylation Regulator

**Figure 1:**
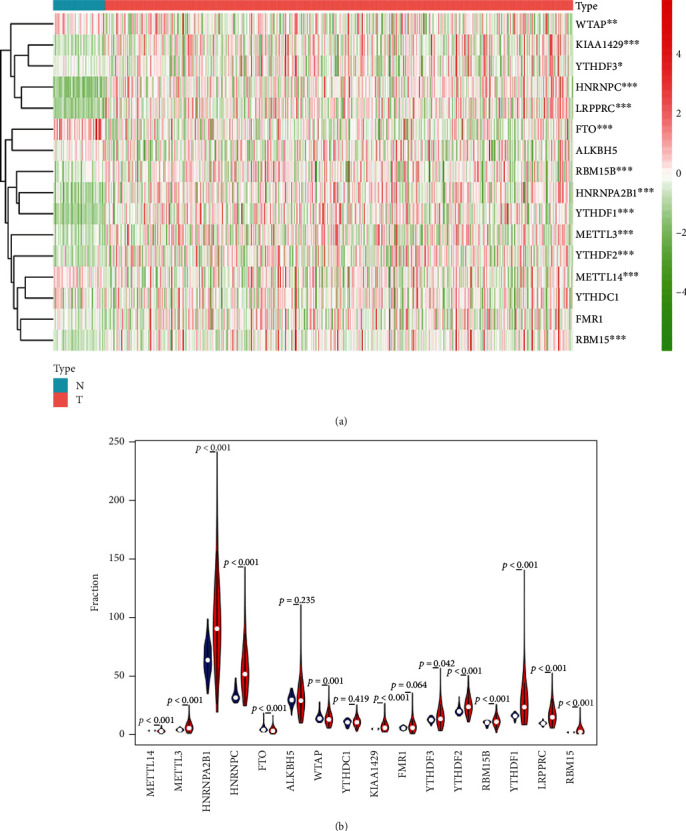
The heat map (a) and violin diagram (b) of differential expression of M6A-related genes in normal samples and lung adenocarcinoma samples. The color from green to red shows a trend from low expression to high expression. Red represents high expression, and blue represents low expression. *P* < 0.05 as the statistical cutoff value.

Based on the transcriptome data associated with LUAD in TCGA, we extracted the transcriptome data of 16 M6A RNA methylation regulatory factors and analyzed their expression in normal persons and in patients with LUAD using the edger software package. The expression of encoders (Vir like M6A methyltransferase associated (KIAA1429), METTL3, putative RNA-binding proteins (RBM)15, and 15B) and readers (leucine-rich PPR motif-containing protein (LRPPRC), heterogeneous nuclear ribonucleoproteins C1/C2 (HNRNPC), YTH domain family, members 1, 2, and 3 (YTHDF1, YTHDF2, and YTHDF3), and heterogeneous nuclear ribonucleoproteins A2/B1 (HNRNPA2B1)) was significantly higher in LUAD, than in normal tissues. Expression of the eliminator, fat mass, and obesity-associated protein (also known as alpha-ketoglutarate-dependent dioxygenase; FTO) and the encoders methyl transferase like 14 (METTL14) and Wilms tumor 1 associated protein (WTAP) were significantly lower in LUAD, than in normal tissues (*P* < 0.05). The expression of readers (YTH domain-containing *protein* 1 (YTHDC1)) and fragile X mental retardation protein (FMR1) and the eliminator, AlkB Homolog 5 (ALKBH5), an RNA demethylase, did not significantly differ between normal and LUAD tissues (*P* > 0.05) (Figure 1). These results suggested that M6A RNA methylation regulators play important roles in the occurrence and progression of LUAD.

### 3.2. Cluster Type of M6A RNA Methylation Regulation Factors Significantly Correlated with Survival of Patients with LUAD

**Figure 2:**
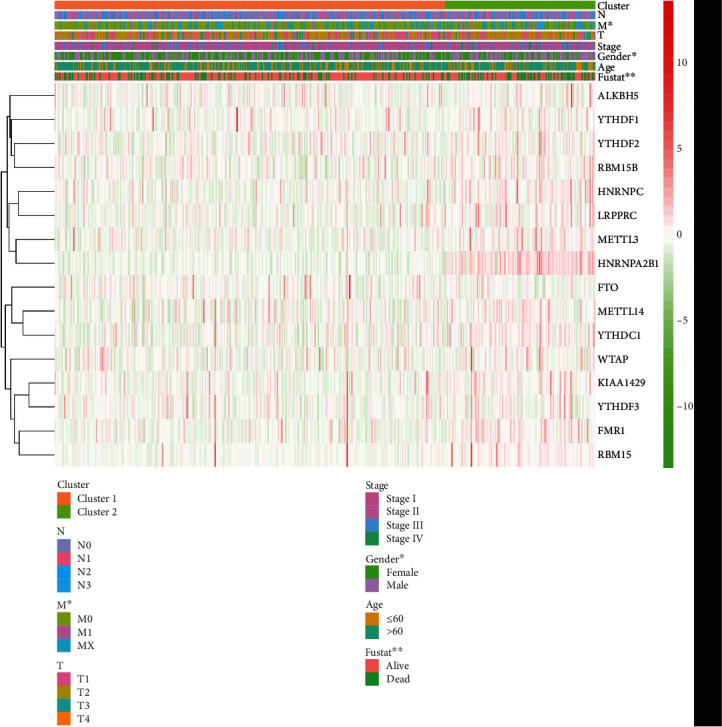
The heat map of clinically associated traits of two clusters. The two clusters significantly differed in terms of sex, M phase, and survival status.

**Figure 3:**
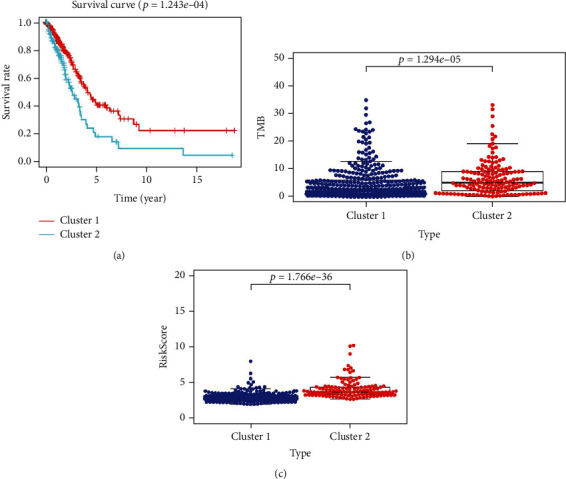
(a) Kaplan-Meier curves of overall survival (OS) for patients with LUAD in two clusters (cluster 1/2). (b) The TMB levels in cluster 1/2 subtypes in TCGA cohort. (c) The risk score in cluster 1/2 subtypes in TCGA cohort.

The results of 16 M6A methylation regulatory factor expression and ConsensusClusterPlus aggregation analysis showed that *K* = 2 had the best cluster stability (*K* = 2–9) (Supplement 1). We divided tissue samples from 535 patients with LUAD into subtypes called clusters 1 (*N* = 382) and 2 (*N* = 153) and analyzed differences between them. Except for eliminators (FTO and ALKBH5), the expression of the other M6A methylation regulators was significantly higher in cluster 2 than 1 (Table 2). When we combined the clinically associated traits, the two subtypes significantly differed in terms of sex, M phase, and survival status (Figure 2). In addition, the Kaplan-Meier curves showed that survival was significantly longer in cluster 1 compared with cluster 2 (Figure 3(a)).

### 3.3. Characteristic Differences in TMB and Immune Cell Infiltration among Cluster Typing

The median numbers of mutations in clusters 1 and 2 TMB were 2.92 (0.02–34.84) and 5.18 (0.21–33.05)/Mb, respectively. And there was significant difference in TMB between the two subtypes (Figure 3(b)).

**Figure 4:**
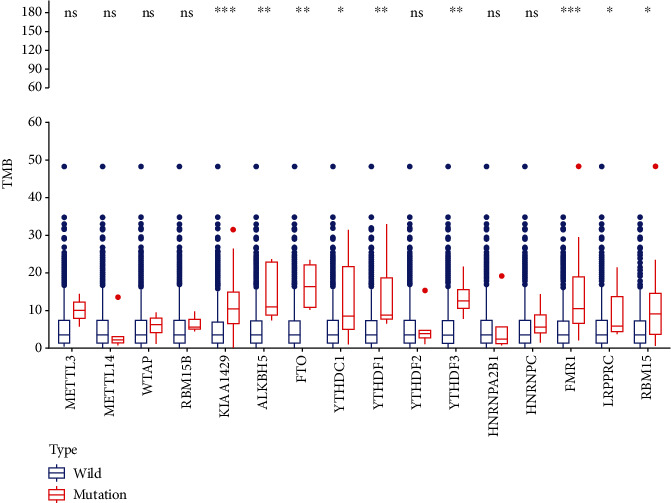
Differences in TMB levels of 16 M6A-related genes between mutant groups and wild groups. Blue represents wild groups, red represents mutant groups, ∗ represents *P* < 0.05, ∗∗ represents *P* < 0.01, and ∗∗∗ represents *P* < 0.001.

**Figure 5:**
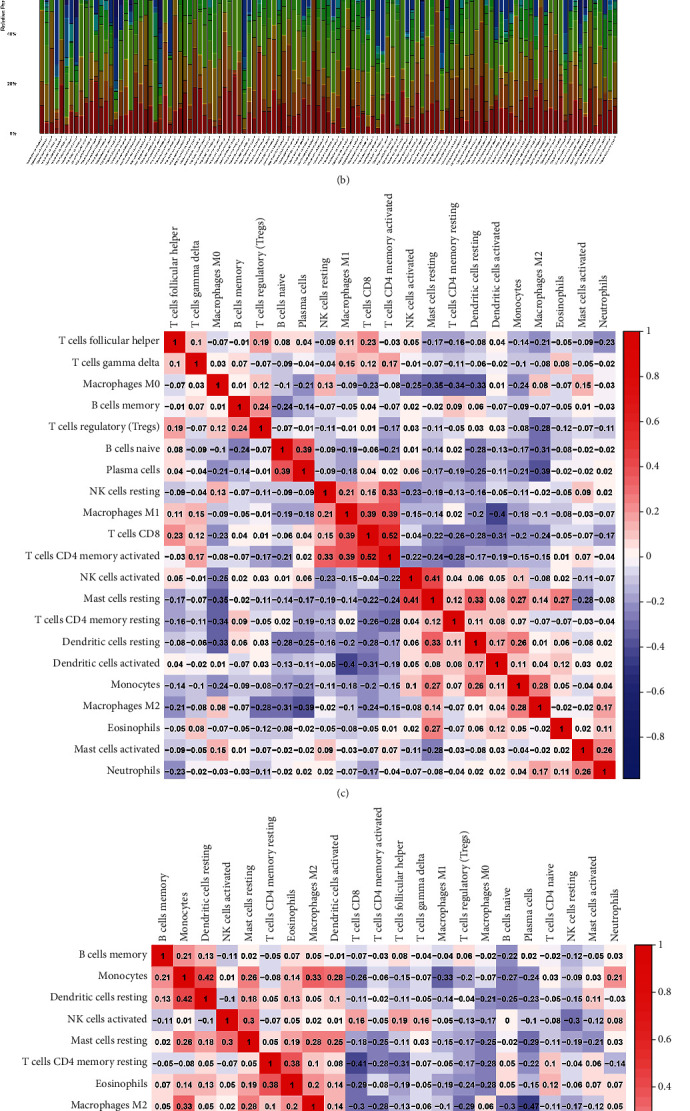
Evaluation of immune cell abundance in different clusters of LUAD samples by CIBERSOFT algorithm. (a) Cluster 1. (b) Cluster 2. Different colors represent different immune cell infiltration. (c) Correlation analysis of immune cell abundance in cluster 1. (d) Correlation analysis of immune cell abundance in cluster 2.

In addition, according to the analysis of LUAD sample data based on 16 M6A methylation regulatory factors, we found that TMB levels significantly differed between the mutant and wild groups. The TMB was higher in the mutant, than the wild group, especially in terms of the regulatory factors KIAA1429 and FMR1 (Figure 4). The abundance of immune cells evaluated using the CIBERSOFT algorithm also differed between clusters 1 and 2, showing that cluster 1 closely correlated with neutrophils, macrophages M2, monocytes, resting dendritic cells, and resting mast cells, whereas cluster 2 was more correlated with M0 and M1 macrophages, follicular helper T cells, resting NK cells, and CD4 memory-activated T cells (Figure 5).

We analyzed gene enrichment using GSEA to clarify potential regulatory mechanisms that might lead to the differences between the two subgroups. Cluster 1 was mainly enriched in arachidonic acid metabolism and the metabolism of drugs and xenobiotics by cytochrome P450, whereas cluster 2 was mainly enriched in cell cycle, as well as MTOR and p53 signaling pathway pathways (Supplement 2), which are closely associated with tumors.

### 3.4. Construction of Risk Scoring System for M6A RNA Methylation Regulator

The univariate Cox regression analysis of the TCGA training cohorts (*n* = 245) showed that HNRNPA2B1 and HNRNPC significantly correlated with patient prognosis (*P* < 0.05; Table 3). Based on the univariate Cox regression results, we created a multivariate model and analyzed the minimum absolute contraction and selection operator (lasso) and multivariate Cox regression. We determined that the regulatory factors HNRNPA2B1 and HNRNPC could be used to construct a risk score system for LUAD. The risk score of each sample was determined based on the coefficient analysis of the two adjustment factors as

(2) $$\begin{array}{c} \left(0.0075\times \text{HNRNPA}2\text{B}1\text{ expression}\right)+\left(0.01424\times \text{HNRNPC expression}\right). \end{array}$$

**Figure 6:**
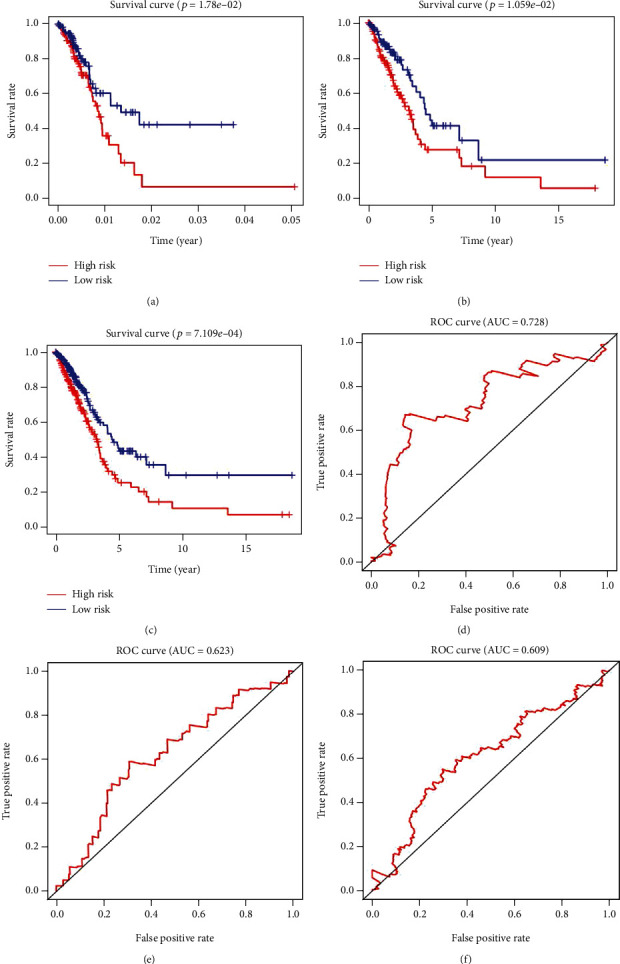
Kaplan-Meier for the risk scoring system based on 2 M6A-related genes (*P* < 0.05 as the statistical cutoff value). (a) TCGA training cohorts. (b) TCGA validation cohorts. (c) TCGA all LUAD samples. Time-dependent ROC curves. (d) TCGA training cohorts. (e) TCGA validation cohorts. (f) TCGA all LUAD samples.

**Figure 7:**
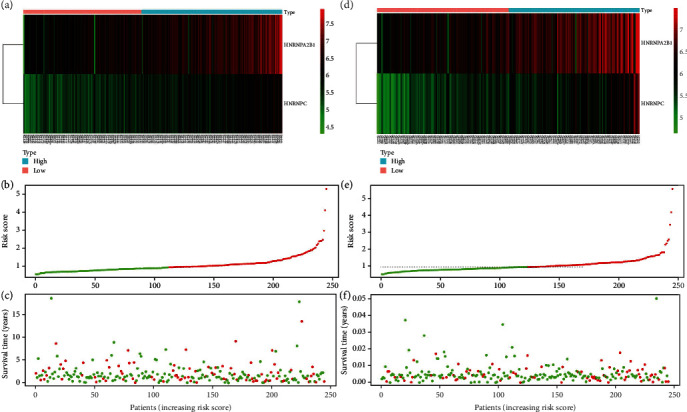
Distribution of risk score, OS, and OS status and heat map of the two prognostic m6A regulator signatures in the TCGA training cohort (a, b, c) and TCGA validation cohort (d, e, f).

Samples from patients were assigned to high- and low-risk groups based on the median risk score. The OS was significantly longer for the low-risk group, than the high-risk group (Figures 6(a)–6(c)). Heat maps showed that more HNRNPA2B1 and HNRNPC tended to be expressed in the high-risk group (Figure 7). We evaluated the predictive accuracy of the risk scoring system by comparing the areas under receiver operator characteristics (ROC) curves (AUC) between the two groups. In the TCGA training cohort, the AUC of the risk score system for predicting 1-year survival was 0.728, and that of the verification set was 0.623. The AUC showed that two M6A regulator factors had good discriminant effects on the short-term prognosis of patients with LUAD (Figures 6(d)–6(f)).

### 3.5. Risk Scoring System as an Independent Prognostic Factor

**Figure 8:**
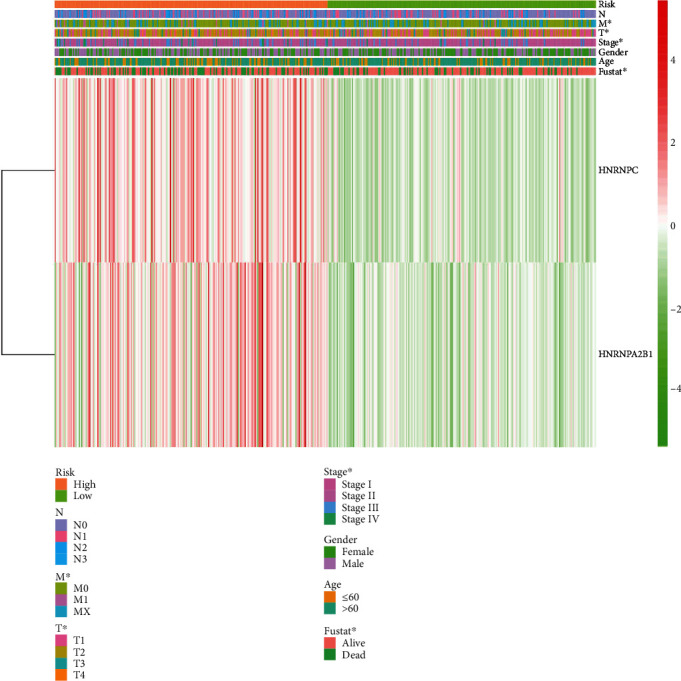
Heat map and clinicopathological features of high- and low-risk groups.

**Figure 9:**
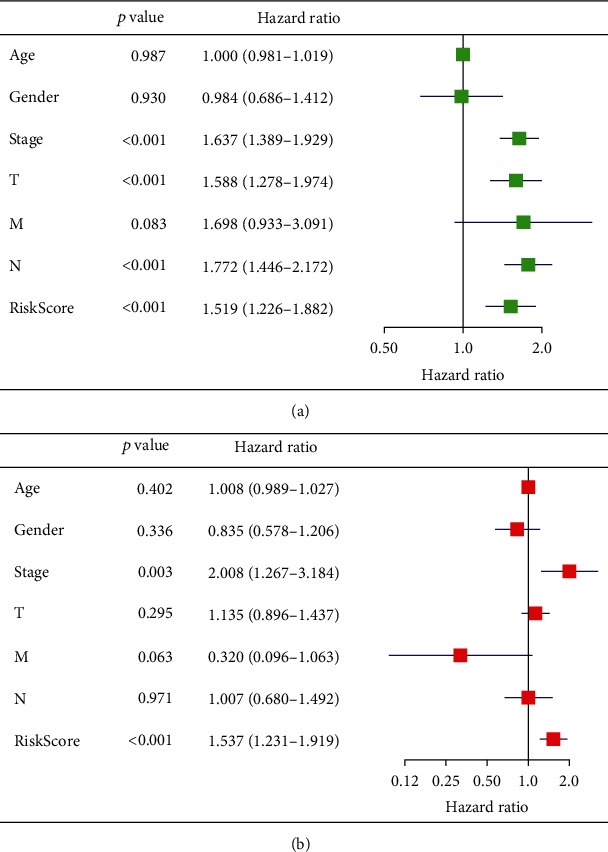
Univariate and multivariate Cox regression of risk score and clinical traits. (a) Univariate Cox regression. (b) Multivariate Cox regression.

The clinical factors associated with LUAD in TCGA were compared between the high- and low-risk groups using heat maps. Stages M and T and fustat all correlated with risk scores, with significant differences between the groups (*P* < 0.05) (Figure 8). The univariate Cox regression showed that risk score and T and N stages were closely associated with OS in patients with LUAD. The multivariate Cox regression showed that the risk score could serve as an independent prognostic factor with which to evaluate patient survival (Figure 9). Thus, the risk scoring system can evaluate the prognosis of patients with LUAD through univariate or multivariate Cox regression analysis. We examined the relationship between risk scores and clustering subtypes and found significantly higher risk scores for cluster 2 than 1 (*P* < 0.001; Figure 3(c)). These results coincided with the finding that OS was longer for cluster 1 than 2 and thus further verified the reliability of typing.

## 4. Discussion

The M6A methylation modification plays an important regulatory role in mRNA metabolism, splicing, translocation, stability, and translation. The regulatory expression and genetic changes of m6A modulators are associated with tumorigenesis, cancer cell proliferation, tumor microenvironment, and cancer prognosis. The modification of M6A methylation can regulate the functions of key downstream genes. Single nucleotide polymorphisms might affect gene expression and biological activity, leading to abnormal downstream m6A-RNA regulation, and subsequently promote tumor initiation and development [23]. In LUAD, M6A regulatory factors participate in the activation of signaling pathways in high-risk patients, such as cell circulation, DNA replication, RNA degradation, RNA polymerase, nucleotide excision repair, and basal transcription factors [24]. Therefore, further study of m6A modulators is needed to clarify potential relationship between m6A methylation and the development and prognosis of LUAD.

We previously evaluated the expression of 16 M6A regulatory-related genes in women with LUAD and the relationship between changes on these genes and clinical characteristics. We then developed a risk scoring system associated with M6A [25]. Compared with other clinical factors, the risk scoring system has high predictive sensitivity and specificity. Here, we further characterized the potential clinical value of m6A to LUAD by comparing differences between 16 m6A modulators between healthy individuals and patients with LUAD and confirmed the roles of the 16 m6A in the occurrence and development of LUAD. Based on the 16 m6A modulators, we further divided 535 patients with LUAD into two subtypes and found that prognosis, sex, M staging, survival status, TMB, immune cell infiltration, and pathways differed between them. We then constructed scoring system comprising HNRNPA2B1 and HNRNPC based on the 16 m6A modulators. This scoring system can serve as an independent prognostic factor for LUAD.

Based on the clinical research value of m6A in the occurrence and development of LUAD, we clustered m6A modulators to characterize the effects of differential m6A methylation modifications on LUAD subtypes. The expression of m6A methylation regulators was lower and OS was significantly longer in cluster 1 than 2. Consistent with previous studies, the abundant expression of m6A modulators might lead to a poor prognosis for patients with LUAD [24]. In addition, sex, M stage, and the survival status of clusters 1 and 2 significantly differed, indicating that sex influences the expression of m6A modulators and that their differential expression can lead to M stage of LUAD. Therefore, using m6A as a clinical marker in patients with LUAD can help clinicians to more comprehensively understand patient status and thus accurately predict, type, and treat LUAD.

Analysis of GSEA enrichment deeply characterized the differences in molecular pathway mechanisms between clusters 1 and 2. Cluster 2 was mainly enriched in the cell cycle, as well as the MTOR, and P53 signaling and other pathways that are closely associated with tumors [26–28]. This might have been involved in the worse prognosis of patients in cluster 2. Compared with cluster 1, which was mainly enriched in metabolism and other pathways, the overactive tumor pathway in cluster 2 was associated with disease progression and predicted survival. We also identified significant differences in the TMB between the m6A subgroups. As a predictive biomarker for the therapeutic efficacy of immune checkpoint inhibitors for various cancer types, TMB is associated with longer OS [29]. The effects on TMB caused by altered m6A modulators might differ among cancer types. The survival and prognosis of subgroups of LUAD patients were related, and the TMB was significantly higher for the mutant, than the wild-type group divided according to the m6A regulators KIAA1429 and FMR1. Immune cell infiltration mediates the immune microenvironment of tumors and is associated tumor occurrence, progression, treatment, and prognosis [30]. Studies of immune cell infiltration in LUAD subtypes revealed differences in the abundance of immune cells between clusters 1 and 2, which might be associated with the difference in survival, and the deeper associated mechanisms require further characterization. Taking m6A modulators as the starting point, exploring the mechanism of intervention in LUAD TMB and immune cell infiltration, which indirectly affects the survival and prognosis of patients, could serve as a potential research model.

The mA regulator signatures KIAA1429, METL3, and IGF2BP1 have been identified as independent prognostic models that can stratify patients, evaluate prognosis, and personalize treatment for lung cancer [31]. Peripheral blood leukocyte m6A can represent a potential noninvasive biomarker for NSCLC screening, monitoring, and diagnosis [32]. Here, we assigned data from 499 patients into training and validation cohorts. Based on 16 m6A modulators, we constructed a LUAD risk scoring system consisting of HNRNPA2B1 and HNRNPC. Previous studies have shown that HNRNP is involved in cancer-related pathways, including protein secretion, mitochondrial spindle, G2/M checkpoint, DNA repair, IL6/JAK/STAT3 signaling, and other pathways. Among them, the range of HNRNPA2B1 copy numbers is wide among various cancer types, and this is associated with the poor survival rate of LUAD [33]. The expression of HNRNPC is abundant in LUAD tissues; it is significantly associated with age, sex, smoking history, race, lymph node metastasis, TNM stages, and low OS rates [34]. Both HNRNPA2B1 and HNRNPC might be cancer-promoting factors and potential prognostic biomarkers for LUAD. Consistent with previous findings, the present study found a trend towards more abundant HNRNPA2B1 and HNRNPC expression in the high-risk group. Moreover, the prognostic risk scoring system discriminated the short-term prognosis of patients with LUAD in the training and verification cohorts.

Clinical prognosis is presently predicted based on clinical and pathological characteristics, including stage, M, T, and fustat. Our risk scoring system comprises HNRNPA2B1 and HNRNPC, and the univariate or multivariate Cox regression analysis can assess the prognosis of patients with LUAD. The risk scoring system correlated with stage, M, T, and fustat in the training and verification cohorts, indicating that the system can specifically and sensitively predict the short-term prognosis of patients with LUAD. The risk scoring system revealed longer OS in cluster 1 than 2, which further verified the clinical effectiveness of the prognostic scoring system. The prognostic risk scoring system can reduce sequencing costs, thus rendering the application of targeted sequencing based on specific genes more cost-effective and routine. However, the current study has some limitations. The quality of samples in the TCGA database was high, but the sample number was quite low. Therefore, our scoring system requires further validation using large-scale clinical data and various regression models to further improve its predictive accuracy.

## 5. Conclusion

In summary, m6A regulatory factors are involved in the molecular mechanism of the occurrence and development of LUAD, and they significantly impact M phase, TMB, immune cell infiltration, molecular pathway network, and survival prognosis. Based on 16 m6A regulatory factors, our risk scoring system consisting of HNRNPA2B1 and HNRNPC has specificity and sensitivity for judging the short-term prognosis of patients with LUAD. Our scoring system can further improve the accuracy of prognostic risk assessment by combining it with clinical evaluation criteria. It also has auxiliary significance for improving the level of diagnosis and quality of treatment for patients with LUAD.

## Figures and Tables

**Table 1:** The specific baseline clinicopathological characteristics of 490 LUAD samples.

	490 LUAD samples
Age	
<60 years	136
≥60 years	354
Stage	
I	261
II	117
III	79
IV	25
Unknown	8
Pathologic T stage	
T1-2	424
T3-4	63
Unknown	3
Pathologic N stage	
N0-1	409
N2-3	70
Unknown	11
Pathologic M stage	
M0	322
M1	24
Unknown	144
Survival time	
≤1 years	127
1 years < ≤3 years	273
3 years < ≤5 years	52
>5 years	38

**Table 2:** Expression levels and differences of 16 M6A methylation regulators in cluster 1 and cluster 2.

Gene	Cluster 1	Cluster 2	logFC	*P* value
YTHDF3	14.609	16.37808	0.164909	0.028612
YTHDF2	24.32579	26.02792	0.097573	0.009162
METTL14	3.200071	3.614964	0.175878	0.001386
WTAP	13.45123	14.93013	0.150489	0.001057
RBM15B	11.3379	12.6848	0.161946	0.000137
YTHDF1	25.2132	28.97565	0.200662	6.88E-08
LRPPRC	15.84726	19.52537	0.301116	1.92E-08
METTL3	6.090176	7.911402	0.377449	3.07E-09
KIAA1429	6.607734	8.094922	0.292862	6.48E-10
HNRNPC	52.3006	60.40718	0.207892	4.98E-10
FMR1	6.356802	8.812112	0.471187	9.89E-13
YTHDC1	10.51121	12.72354	0.275572	8.52E-13
RBM15	2.821307	4.000656	0.503873	4.75E-20
HNRNPA2B1	79.92151	127.5456	0.674358	3.28E-72

**Table 3:** Univariate Cox regression analysis of the TCGA training cohorts.

ID	HR	HR.95L	HR.95H	*P* value
METTL14	1.003652	0.762947	1.320298	0.979213
WTAP	1.001162	0.942224	1.063786	0.97008
METTL3	1.003207	0.933483	1.07814	0.93057
ALKBH5	0.998704	0.972253	1.025876	0.924585
YTHDF2	1.005917	0.970619	1.042499	0.746151
YTHDF1	1.003796	0.98692	1.020959	0.661423
FMR1	0.977839	0.891574	1.07245	0.634364
FTO	0.944817	0.807647	1.105284	0.47818
YTHDC1	1.036435	0.949883	1.130874	0.421197
YTHDF3	1.016342	0.977513	1.056713	0.414722
RBM15B	1.041751	0.972685	1.115721	0.242534
KIAA1429	1.055824	0.985611	1.131038	0.121826
LRPPRC	1.02272	0.995493	1.050691	0.102706
RBM15	1.116559	0.993123	1.255338	0.065108
HNRNPA2B1	1.008036	1.000001	1.016136	0.049973
HNRNPC	1.013535	1.002257	1.02494	0.018526

## Data Availability

The data is public and can be downloaded from the TCGA database for free.

## Supplementary Materials

Supplementary 1 Supplement 1: the results of ConsensusClusterPlus aggregation analysis showed that *K* = 2 had the best cluster stability (*K* = 2–9).

Supplementary 2 Supplement 2: gene enrichment of cluster 1 and cluster 2 based on GSEA analysis.
