# Supplementary material for: Comprehensive Analysis of Gene Signatures of m6ARNA Methylation Regulators in Lung Adenocarcinoma and Development of a Risk Scoring System
Source: J Immunol Res. 2022 Aug 23;2022:7519838. doi: 10.1155/2022/7519838 (PMC9428682; doi:10.1155/2022/7519838)
Supplement: Supplementary 1 — Supplement 1: the results of ConsensusClusterPlus aggregation analysis showed that K = 2 had the best cluster stability (K = 2–9). [file 7519838.f1.docx]

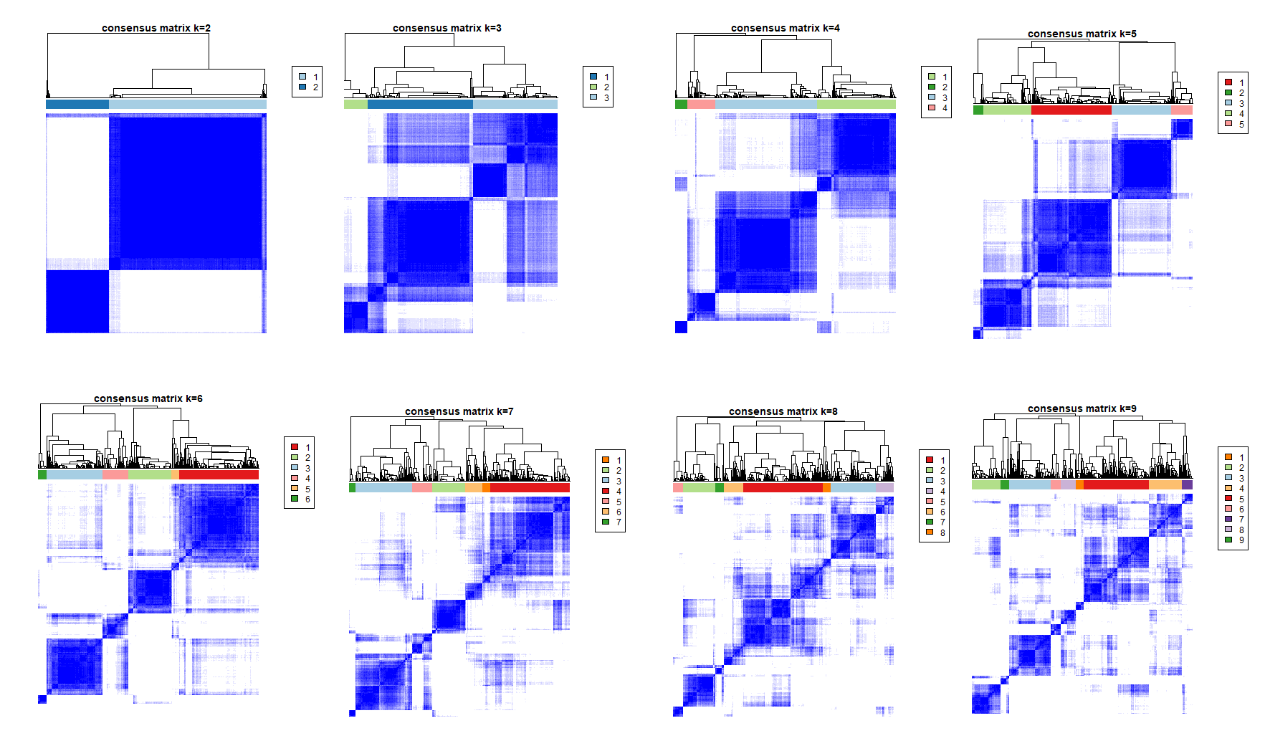


Supplement 1: The results of ConsensusClusterPlus aggregation analysis showed that K = 2 had the best cluster stability (K = 2–9)
