# Supplementary material for: Comprehensive Analysis of Gene Signatures of m6ARNA Methylation Regulators in Lung Adenocarcinoma and Development of a Risk Scoring System
Source: J Immunol Res. 2022 Aug 23;2022:7519838. doi: 10.1155/2022/7519838 (PMC9428682; doi:10.1155/2022/7519838)
Supplement: Supplementary 2 — Supplement 2: gene enrichment of cluster 1 and cluster 2 based on GSEA analysis. [file 7519838.f2.docx]

|  | Pathways | ES | NES | NOM p-val |
| --- | --- | --- | --- | --- |
| Cluster1 | KEGG_ARACHIDONIC_ACID_METABOLISM | 0.652544 | 2.170498 | 0 |
|  | KEGG_DRUG_METABOLISM_CYTOCHROME_P450 | 0.656275 | 2.024395 | 0 |
|  | KEGG_METABOLISM_OF_XENOBIOTICS_BY_CYTOCHROME_P450 | 0.640882 | 1.938656 | 0.001709 |
|  | KEGG_COMPLEMENT_AND_COAGULATION_CASCADES | 0.666029 | 2.066948 | 0.001764 |
|  | KEGG_LYSOSOME | 0.601827 | 1.993689 | 0.001927 |
|  | KEGG_HEMATOPOIETIC_CELL_LINEAGE | 0.623041 | 1.975089 | 0.003704 |
|  | KEGG_ASTHMA | 0.790501 | 1.940567 | 0.005693 |
|  | KEGG_GLYCOSAMINOGLYCAN_DEGRADATION | 0.637011 | 1.804771 | 0.005837 |
|  | KEGG_GLYCOSPHINGOLIPID_BIOSYNTHESIS_GANGLIO_SERIES | 0.656968 | 1.749661 | 0.007435 |
|  | KEGG_INTESTINAL_IMMUNE_NETWORK_FOR_IGA_PRODUCTION | 0.709322 | 1.884559 | 0.009488 |
|  | KEGG_RIBOSOME | 0.884615 | 1.832644 | 0.013645 |
|  | KEGG_RETINOL_METABOLISM | 0.52715 | 1.639415 | 0.022436 |
|  | KEGG_PROXIMAL_TUBULE_BICARBONATE_RECLAMATION | 0.553117 | 1.588109 | 0.027244 |
|  | KEGG_ETHER_LIPID_METABOLISM | 0.476041 | 1.557239 | 0.031079 |
|  | KEGG_CELL_ADHESION_MOLECULES_CAMS | 0.527931 | 1.731895 | 0.037523 |
|  | KEGG_ALLOGRAFT_REJECTION | 0.702967 | 1.64331 | 0.038685 |
|  | KEGG_ALDOSTERONE_REGULATED_SODIUM_REABSORPTION | 0.453174 | 1.545766 | 0.041145 |
| Cluster 2 | KEGG_CELL_CYCLE | -0.7014 | -2.26623 | 0 |
|  | KEGG_OOCYTE_MEIOSIS | -0.5788 | -2.20186 | 0 |
|  | KEGG_RNA_DEGRADATION | -0.65925 | -2.18705 | 0 |
|  | KEGG_HOMOLOGOUS_RECOMBINATION | -0.80407 | -2.11874 | 0 |
|  | KEGG_DNA_REPLICATION | -0.87959 | -2.09412 | 0 |
|  | KEGG_MISMATCH_REPAIR | -0.82546 | -2.07342 | 0 |
|  | KEGG_NUCLEOTIDE_EXCISION_REPAIR | -0.65132 | -1.99464 | 0 |
|  | KEGG_UBIQUITIN_MEDIATED_PROTEOLYSIS | -0.48283 | -1.90582 | 0.001996 |
|  | KEGG_SPLICEOSOME | -0.71146 | -2.2061 | 0.002041 |
|  | KEGG_PROGESTERONE_MEDIATED_OOCYTE_MATURATION | -0.54748 | -2.03574 | 0.002066 |
|  | KEGG_LYSINE_DEGRADATION | -0.55853 | -1.80641 | 0.006224 |
|  | KEGG_BASE_EXCISION_REPAIR | -0.6582 | -1.86046 | 0.00813 |
|  | KEGG_PURINE_METABOLISM | -0.41606 | -1.68374 | 0.010823 |
|  | KEGG_PYRIMIDINE_METABOLISM | -0.50607 | -1.77145 | 0.012448 |
|  | KEGG_BASAL_TRANSCRIPTION_FACTORS | -0.56785 | -1.79296 | 0.018789 |
|  | KEGG_PROSTATE_CANCER | -0.4157 | -1.59195 | 0.019355 |
|  | KEGG_MTOR_SIGNALING_PATHWAY | -0.42372 | -1.59927 | 0.027426 |
|  | KEGG_ONE_CARBON_POOL_BY_FOLATE | -0.64339 | -1.74564 | 0.027944 |
|  | KEGG_P53_SIGNALING_PATHWAY | -0.42571 | -1.61961 | 0.0375 |
